# New Parvovirus Associated with Serum Hepatitis in Horses after Inoculation of Common Biological Product

**DOI:** 10.3201/eid2402.171031

**Published:** 2018-02

**Authors:** Thomas J. Divers, Bud C. Tennant, Arvind Kumar, Sean McDonough, John Cullen, Nishit Bhuva, Komal Jain, Lokendra Singh Chauhan, Troels Kasper Høyer Scheel, W. Ian Lipkin, Melissa Laverack, Sheetal Trivedi, Satyapramod Srinivasa, Laurie Beard, Charles M. Rice, Peter D. Burbelo, Randall W. Renshaw, Edward Dubovi, Amit Kapoor

**Affiliations:** Cornell University, Ithaca, New York, USA (T.J. Divers, B.C. Tennant, S. McDonough, M. Laverack, R.W. Renshaw, E. Dubovi);; Nationwide Children’s Hospital, Columbus, Ohio, USA (A. Kumar, S. Trivedi, S. Srinivasa, A. Kapoor);; North Carolina State University, Raleigh, North Carolina, USA (J. Cullen);; Columbia University, New York, New York, USA (N. Bhuva, K. Jain, L.S. Chauhan, W.I. Lipkin);; University of Copenhagen, Copenhagen, Denmark (T.K.H. Scheel); Hvidovre Hospital, Copenhagen (T.K.H. Scheel);; Rockefeller University, New York (T.K.H. Scheel, C.M. Rice);; Kansas State University, Manhattan, Kansas, USA (L. Beard);; National Institute of Dental and Craniofacial Research, National Institutes of Health, Bethesda, Maryland, USA (P.D. Burbelo)

**Keywords:** parvovirus, hepatitis, horse, veterinary virology, metagenomics, equine liver disease, animal model, Theiler’s disease, biologics, viruses, United States

## Abstract

Equine serum hepatitis (i.e., Theiler’s disease) is a serious and often life-threatening disease of unknown etiology that affects horses. A horse in Nebraska, USA, with serum hepatitis died 65 days after treatment with equine-origin tetanus antitoxin. We identified an unknown parvovirus in serum and liver of the dead horse and in the administered antitoxin. The equine parvovirus-hepatitis (EqPV-H) shares <50% protein identity with its phylogenetic relatives of the genus *Copiparvovirus*. Next, we experimentally infected 2 horses using a tetanus antitoxin contaminated with EqPV-H. Viremia developed, the horses seroconverted, and acute hepatitis developed that was confirmed by clinical, biochemical, and histopathologic testing. We also determined that EqPV-H is an endemic infection because, in a cohort of 100 clinically normal adult horses, 13 were viremic and 15 were seropositive. We identified a new virus associated with equine serum hepatitis and confirmed its pathogenicity and transmissibility through contaminated biological products.

Equine serum hepatitis (i.e., Theiler’s disease or idiopathic acute hepatitis) is a serious and often life-threatening disease of horses that was first described in 1919 in South Africa by Sir Arnold Theiler. Theiler observed hundreds of cases of a highly fatal form of hepatitis after experimental vaccination studies to prevent African horse sickness during which infectious virus was administered simultaneously with convalescent equine antiserum (*1*). The incidence of fulminant hepatitis among horses receiving antiserum in outbreaks of Theiler’s disease has been reported to be 1.4%–2.2% (*1*,*2*). Theiler’s disease has been described in horses in many areas of the world after treatment with a variety of equine serum products, including tetanus antitoxin (*3*–*8*), botulinum antitoxin (*9*), antiserum against *Streptococcus equi* (*4*,*10*), pregnant mare’s serum (*4*), and equine plasma (*1*,*2*,*5*,*11*). The clinical disease has a high rate of death, but some horses survive, and survivors have not been reported to have evidence of persistent liver disease (*5*,*8*).

Recently, 3 new flaviviruses were identified in horses (*9*,*12*,*13*). The first was the nonprimate hepacivirus (NPHV), later called equine hepacivirus (*2*,*12*), which is most closely related to hepatitis C virus (*14*). Natural NPHV infection in horses is reported to cause temporary elevation in liver enzymes, and negative-strand viral RNA was detected within hepatocytes (*15*–*17*). The 2 other flaviviruses of horses are Theiler’s disease–associated virus (TDAV) and equine pegivirus (EPgV), both members of the *Pegivirus* genus (*14*). TDAV was identified during an outbreak of acute clinical hepatitis in horses, 6 weeks after prophylactic administration of botulinum antitoxin of equine origin (*9*). EPgV is reported to be a common infection of horse populations of the United States and Western Europe, is not hepatotropic, and has not been associated with hepatic disease (*13*,*16*,*18*).

We identified a new parvovirus in the serum and liver of a horse that died in Nebraska, USA; the virus was also present in the tetanus antitoxin administered to the horse 65 days before disease onset. We acquired the complete viral genome from that horse and, considering its phylogenetic analysis, tentatively named the virus equine parvovirus hepatitis (EqPV-H). We describe the discovery of EqPV-H, its complete genome, infection prevalence, and virus transmission by inoculation of a commercial equine serum product resulting in hepatitis, thereby confirming EqPV-H association with equine hepatitis.

## Materials and Methods

### Sample Collection from the Index Horse

On November 6, 2013, a horse living in Nebraska, USA, was treated prophylactically with tetanus antitoxin (manufactured in Colorado, USA) 65 days before the onset of clinical signs of liver failure. After the horse died of Theiler’s disease in January 2014, serum and liver samples were collected and frozen or kept on ice before being shipped from the clinic of origin to Cornell University (Ithaca, NY, USA) for viral diagnostic testing. The remaining antitoxin in the vial that had been administered to the horse was shipped on ice for virologic testing. Aliquots of lots of commercial tetanus antitoxin to be used in the experimental inoculation were tested by PCR for EqPV-H, TDAV, NPHV, and EPgV as described previously (*9*,*12*,*13*).

### Unbiased Amplification and High-Throughput Sequencing

We prepared liver suspension using ≈100 mg of liver tissue in 1 mL of phosphate-buffered saline and 3-mm steel beads using tissue lyser (QIAGEN, Hilden, Germany). We centrifuged liver suspension and the antitoxin at 5,000 rpm for 10 min to remove the cell debris and filtered clarified supernatant through a 0.45-μ filter (Millipore, Burlington, MA, USA) and treated with nucleases to digest free nucleic acids for enrichment of viral nucleic acid. We performed sequencing library preparation, sequencing, and bioinformatics as described previously (*19*).

### Complete Genome Sequencing and Genetic Analysis of EqPV-H

**Figure 1:**
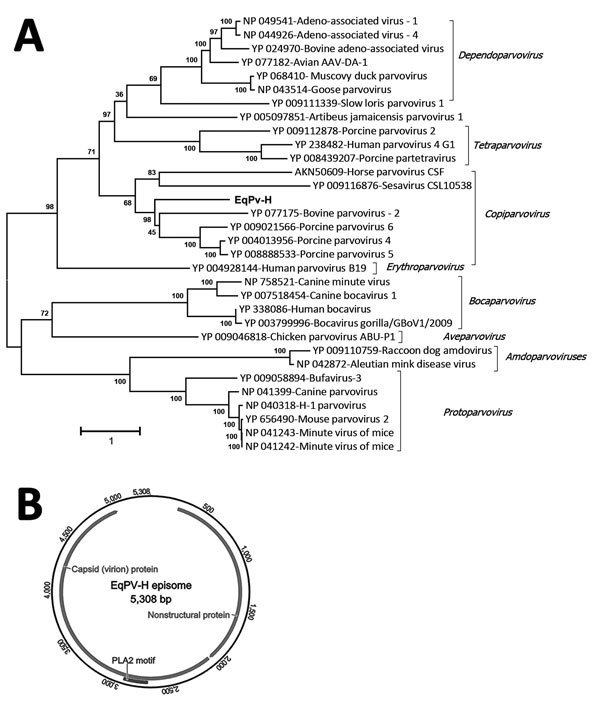
Analysis of equine parvovirus genome. A) Phylogenetic tree showing relationship of EqPV-H to known parvoviruses in the nonstructural protein. GenBank accession numbers are provided. Scale bar indicates amino acid substitutions per site. B) Genomic organization of the EqPV-H episome (*24*). CSF, cerebrospinal fluid; EqPV-H, equine parvovirus hepatitis.

We acquired the complete genome of EqPV-H using a primer walking approach as previously described by us for several new animal parvoviruses, including bocaviruses (*20*–*22*). The genome of EqPV-H episome is available in GenBank (accession no. MG136722). To determine the sequence relationship between EqPV-H and other known parvovirus species, we used >1 representative virus member, including the reference genome from each species and its translated protein sequences, to generate sequence alignments. We generated a phylogenetic tree showing sequences used for the comparison and their GenBank accession numbers (Figure 1, panel A). The evolutionary history was inferred by using the maximum-likelihood method based on the Le_Gascuel_2008 model (*23*). The tree with the highest log likelihood is shown. We conducted evolutionary analyses in MEGA (*24*). DNA secondary structures of genomic termini were predicted by Mfold (*25*).

### PCR and Serologic Assays for EqPV-H

We aligned the nonstructural (NS) protein and virion protein (VP) of EqPV-H to all known parvovirus proteins. We used nucleotide and amino acid motifs showing relative conservation among different virus lineages to make primers for screening samples for EqPV-H and related variants. All PCR mixtures used AmpliTaq Gold 360 master mix (catalog no. 4398881; Applied Biosystems, Foster City, CA, USA) and 2 μL of extracted nucleic acids. The EqPV-H NS gene PCR used primer pair EqPV ak1 (5′-GGAGAAGAGCGCAACAAATGCA-3′) and EqPV ak2 (5′-AAGACATTTCCGGCCGTGAC-3′) in the first round of PCR and the pair EqPV ak3 (5′-GCGCAACAAATGCAGCGGTTCGA-3′) and EqPV ak4 (5′-GGCCGTGACGACGGTGATATC-3′) in the second round of PCR. The EqPV-H VP gene PCR used primer pair EqPV ak5 (5′-GTCGCTGCATTCTGAGTCC-3′) and EqPV ak6 (5′-TGGGATTATACTGTCTACGGGT-3′) in the first round of PCR and the pair EqPV ak7 (5′-CTGCATTCTGAGTCCGTGGCC-3′) and EqPV ak8 (5′-CTGTCTACGGGTATCCCATACGTA-3′) in the second round of PCR.

### Luciferase Immunoprecipitation System Assay for EqPV Serology

We cloned the C terminus of the EqPV-H capsid protein into pREN2 plasmid for making Renilla luciferase fused antigen for a Luciferase Immunoprecipitation System (LIPS) assay. In brief, we amplified the VP1 gene of EqPV-H using primers EqPV LIPSF1 (5′-AGTAAAGTCAATGGACACCA-3′) and EqPV LIPSR1 (5′-GGATCGTGGTATGAGTTC-3′). We sequenced PCR product and then used it as a template to make inserts for LIPS assay using primers with flanking restriction sites, EqPV LIPS *Bam*HI (5′-GAGGGATCCCATGCTTTACCGTATGATC-3′) and EqPV-H LIPS XhoI (5′-GAGCTCGAGTCAGAACTGACAGTATTGGTTC-3′. Inserts were subsequently sequenced, digested, and ligated into pREN-2 expression vector. Details of LIPS antigen preparation and serologic testing have been described previously (*12*,*26*–*28*).

### Experimental Infection of Horses

We selected 2 healthy 18- and 20-year-old mares from the Cornell University College of Veterinary Medicine teaching and research herd that were negative by PCR for EqPV-H, TDAV, and NPHV. We inoculated the horses 4 months apart with 2 lots of tetanus antitoxin that were PCR positive for EqPV-H. These sample lots were selected because they had been reported to us to have been associated with additional cases of Theiler’s disease. We pooled both lots for inoculation so that each horse received the identical inoculum. In each experimental horse, we administered 5.0 mL of the pooled tetanus antitoxin intravenously and 5.0 mL subcutaneously in the neck. We collected blood samples for biochemical analysis in the experimental inoculation study from the jugular vein into 7-mL sodium heparin tubes (BD Vacutainer; Becton Dickinson, Franklin Lakes, NJ, USA) and promptly submitted them to the New York State Animal Health Diagnostic Center at Cornell University for biochemical tests indicative of hepatic disease: aspartate aminotransferase (AST), sorbitol dehydrogenase (SDH), γ-glutamyltransferase (GGT), total bile acids, and bilirubin. Tests were performed using a Hitachi Mod P 800 (Roche Diagnostics, Indianapolis, IN, USA).

We performed liver biopsies after sedation with 5 mg detomidine administered intravenously; local anesthetic (2% lidocaine) was injected subcutaneously at the planned insertion site of the percutaneous biopsy instrument (Tru-Cut Biopsy Needle; Travenol Laboratories, Deerfield, IL., USA). We determined the location of the biopsy needle insertion by ultrasonographic visualization of the liver. We placed samples in formalin for histopathologic studies and submitted fresh liver tissue to the same laboratory for quantitative PCR. The case–control and experimental inoculation studies were approved in full by the Cornell University Animal Research Committee (IACUC no. 2014-0024).

## Results

### Identification of EqPV-H in Horse with Theiler’s Disease

Serum and liver samples from the deceased horse obtained postmortem and from the administered tetanus antitoxin tested negative for the 3 recently identified horse flaviviruses, NPHV, TDAV, and EPgV (*29*). We used an unbiased amplification and high-throughput sequencing approach to identify known and new viruses in the antitoxin and liver sample (*19*,*23*,*30*). Bioinformatics of sequence data revealed a 4.5-kb base assembled sequence in the horse liver sample that showed distant yet significant protein similarity with known bovine and porcine parvoviruses. Thereafter, the in silico assembled sequence data were used to design primers for amplifying the complete genome of the new parvovirus, tentatively named EqPV-H. Considering the presence of high virus titer in the liver sample and the precedent of finding virus episomes in tissue for related parvoviruses (*20*), we used an inverse PCR–based approach to acquire the complete virus genome. The inverse PCR confirmed that EqPV-H exists as episomes in the liver tissue.

**Figure 2:**
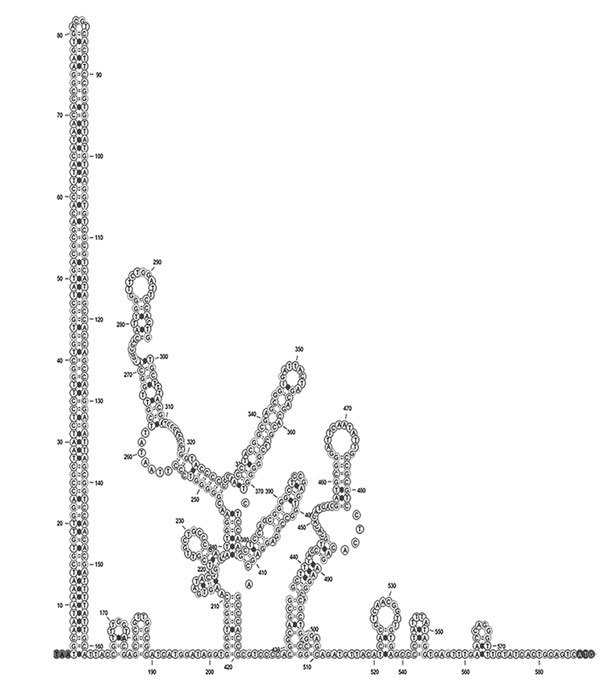
EqPV-H DNA secondary structure of the 583-nt intergenic region predicted using mFOLD (*25*). EqPV-H, equine parvovirus hepatitis.

The complete episome of EqPV-H comprises 5,308 nt and is predicted to code 2 large open reading frames whose proteins are related to the NS proteins and the structural proteins (VPs) of known animal parvoviruses (Figures 1, 2). If the first nucleotide of NS protein is considered to be genome position 1, the NS protein is coded by nucleotide positions 1–1,779, followed by an intergenic region of 21 nt, and the VPs are coded by nucleotide positions 1,801–4,722. An intergenic region of 583 nt connects the end of VP coding region to the NS coding region in the episomal genome form of EqPV-H. The genomic termini of parvoviruses play an important role in virus replication and translation. Similar to known animal parvoviruses, the DNA folding programs predicted 1 long hairpin and other small hairpins in the intergenic region of EqPV-H (Figure 2). Attempts to propagate the virus in cell culture using the serum and liver samples of infected horses have been unsuccessful.

EqPV-H genome organization and genetic relatedness to known viruses suggest its classification as a prototype of new species in the genus *Copiparvovirus* (Figure 1, panel A). Other members of the genus *Copiparvovirus* include parvoviruses that infect pigs, cows, and sea lions and a recently identified virus found in horse cerebrospinal fluid (CSF), horse parvovirus–CSF (*31*). Since the NS proteins of parvoviruses are relatively more conserved than the VP, we used the NS protein of EqPV-H for its phylogenetic analysis. Results indicate that EqPV-H is distinct, yet most closely related to different species of copiparvoviruses (Figure 1, panel A). The EqPV-H is more closely related to pig and cow copiparvovirus than to the only other horse copiparvovirus, indicating different evolutionary origins of these 2 horse viruses. This NS gene–based phylogenetic analysis was further confirmed using VP gene–based phylogenetic analysis (data not shown).

### Infection Prevalence, Disease Association, and Molecular Epidemiology

To determine the infection prevalence of EqPV-H, we tested 100 horse serum samples of convenience submitted to the New York State Animal Health Diagnostic Center at Cornell University for nonclinical reasons. PCRs targeting the NS and VP region of EqPV-H identified 13 of 100 horses positive for EqPV-H viremia. We also tested these samples for EqPV-H IgG using partial VP1 as antigen in the LIPS assay. We determined that all 13 viremic horses had IgG. In addition, 2 nonviremic horses were seropositive, indicating clearance of EqPV-H viremia. We then tested the 13 virus-positive samples biochemically for evidence of liver disease using GGT as a marker; all results were within normal range. Genetic analysis of EqPV-H variants found in all the studied serum samples indicated a very low level of genetic diversity (<2% nt differences in NS and VP sequences) among isolates.

### Experimental Inoculation of Commercial Tetanus Antitoxin

**Figure 3:**
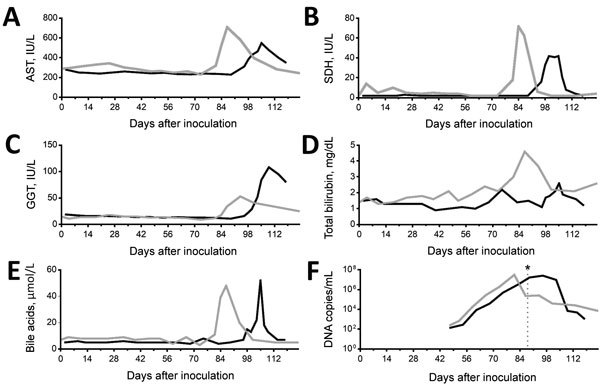
Kinetics of viremia and liver enzymes and time of seroconversion in 2 horses experimentally inoculated with equine parvovirus hepatitis. Gray line indicates horse 1; black line indicates horse 2. A) AST; B) SDH; C) GGT; D) total bilirubin; E) bile acids; F) DNA copies. Asterisk indicates time of seroconversion. AST, aspartate aminotransferase; GGT, γ-glutamyltransferase. SDH, sorbitol dehydrogenase.

To confirm transmission of EqPV-H by tetanus antitoxin, we inoculated 2 clinically normal mares with 10 mL of tetanus antitoxin positive for EqPV-H. The 2 horses were confirmed PCR negative for EqPV-H, TDAV, and NPHV nucleic acids and LIPS negative for EqPV-H antibody. At weekly intervals after inoculation, we tested both experimentally inoculated horses for NPHV, TDAV, and EqPV-H nucleic acids by quantitative PCR, for EqPV-H antibodies by LIPS, and for biochemical evidence of liver disease (Figure 3). Both horses remained PCR negative for EqPV-H nucleic acids on weekly sampling until 47 and 48 days postinoculation (dpi), at which time both horses became PCR positive for EqPV-H. Consecutive weekly samples demonstrated increasing viremia in both horses; viremia peaked at 81 and 96 dpi. Thereafter, viremia gradually decreased, but EqPV-H was still detected in the serum of both horses at study termination (123 and 125 dpi). Antibodies against EqPV-H capsid protein were first detected at 88 and 89 dpi in the 2 infected horses. Both horses remained PCR negative for NPHV and TDAV at study termination.

**Figure 4:**
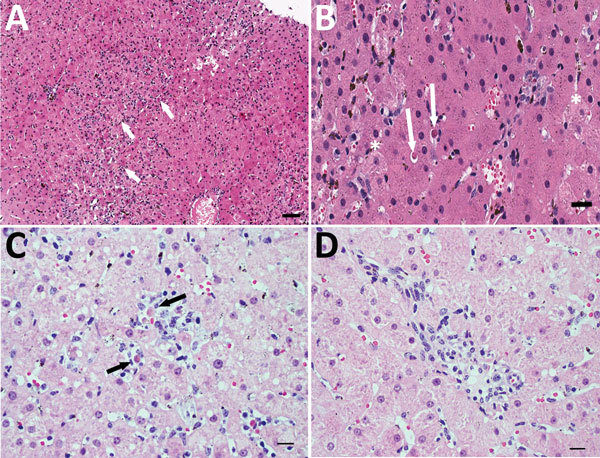
Histopathologic findings in the livers of 2 adult horses experimentally infected with an equine biological product containing equine parvovirus-hepatitis (EqPV-H). A) Liver biopsy sample from horse 1 obtained 82 days after inoculation with EqPV-H. Numerous individual and small clusters of lymphocytes are scattered about the parenchyma (arrows), indicative of lymphocytic lobular hepatitis. Hematoxylin and eosin (H&E) stain. Scale bar = 200 μm. B) Higher magnification image of the liver biopsy sample illustrated in panel A. Two individual necrotic hepatocytes are highlighted (arrows). Note the shrunken cell bodies, hypereosinophilic cytoplasm, and pyknotic nucleus, compatible with acidophil bodies. Other cells (asterisks) are swollen, with pale, mildly vacuolated cytoplasm, interpreted as hydropic degeneration. The cellular pleomorphism resulted in a mild degree of lobular disarray. Kupffer cells on the left side of the image contain hemosiderin, which is normal in horses. H&E stain. Scale bar = 50 μm. C) Liver biopsy sample from horse 2 obtained 100 days after inoculation with EqPV-H. Lymphocytes (black arrows) surround individual and small clusters of necrotic hepatocytes. Lymphocytic satellitosis implicates immune-mediated killing of hepatocytes by cytotoxic lymphocytes. H&E stain. Scale bar = 50 μm. D) Liver biopsy sample from horse 2. The portal tracts are infiltrated by small numbers of lymphocytes that breach the limiting plate and obscure the boundaries. No piecemeal necrosis (i.e., individual hepatocyte necrosis in the limiting plate) is detected. H&E stain. Scale bar = 50 μm.

In horse 1, serum biochemical evidence of liver disease was first observed at 82 dpi, when all test parameters except bile acids and total bilirubin showed marked increases (Figure 3). Samples were then tested daily or every other day; bile acids and bilirubin were increased at 84 dpi (Figure 3). At 87 dpi, horse 1 became icteric and depressed and developed orange urine (bilirubinuria). Abnormalities in biochemical parameters increased further until 90 dpi, after which values started to return to normal. Values were normal by 98 dpi except for GGT, which returned to normal range at 114 dpi. Clinical icterus and discolored urine were not observed after 94 dpi. A liver biopsy sample obtained at 82 dpi showed lymphocytic lobular atrophy (Figure 4, panel A) and necrotic hepatocytes (Figure 4, panel B). 

Horse 2 first showed abnormal serum biochemistry values at 96 dpi; AST, SDH, GGT, bile acids, and total bilirubin increased gradually until 105 dpi, when all biochemical values began decreasing and were normal by 118 dpi, except for GGT, which remained mildly elevated at the conclusion of the study (123 dpi). A liver biopsy sample obtained at 100 dpi had lymphocytes surrounding clusters of necrotic hepatocytes and lymphocytic satellitosis (Figure 4, panel C). Portal tracts were also infiltrated with small numbers of lymphocytes that breached the limiting plate (Figure 4, panel D).

## Discussion

The previously unidentified horse parvovirus EqPV-H we describe represents the prototype of a new virus species of genus *Copiparvovirus*. Although a new parvovirus in the genus *Copiparvovirus* was recently found in a CSF sample of a horse with neurologic signs, EqPV-H is genetically very distinct from that virus (*31*). EqPV-H is genetically more similar to other ungulate copiparvoviruses than the horse parvovirus–CSF. Parvoviruses are ubiquitous and are proposed to have a wide range of effects on their hosts, ranging from severe disease to nonpathogenic infections (*32*). The pathogenesis of parvoviruses is generally associated with their predilection for actively dividing cells and different parvoviruses have different organ tropism (*32*). Parvovirus hepatitis in other species is rare, although parvovirus B19 can be associated with acute hepatitis in humans after transfusion of contaminated blood products (*33*,*34*).

Although the overall incidence of clinically recognized serum hepatitis in adult horses receiving tetanus antitoxin is low, tetanus antitoxin has been the most commonly reported blood product associated with the disease in the United States for the past 50 years (*3*,*4*,*6*–*8*). Commercial tetanus antitoxin is heat treated (60°C for 1 h) to inactivate virus, and phenol and thimeresol are added as preservatives. Such treatments could leave detectable viral nucleic acids that would be nontransmissible. However, the 2 successful transmissions of EqPV-H to experimentally inoculated horses confirmed that EqPV-H can be transmitted from heat-treated commercially available tetanus antitoxin. Although this form of heat treatment is known to inactivate heat-labile viruses, such as lentiviruses (*35*), in blood products, parvoviruses and especially animal parvoviruses are very resistant to heat inactivation and solvent detergent treatments (*35*–*38*).

The incubation period for onset of biochemical disease after experimental inoculation of PCR-positive tetanus antitoxin to the 2 horses was longer (82 and 94 days) than most reported clinical cases of equine serum hepatitis. Clinical cases typically occur 6–10 weeks after blood product inoculation, but cases have been reported as long as 14 weeks after blood product administration (*5*,*7*). Complete recovery of both horses in the experimental study was not surprising because rapid (3–7 days) recovery occurs in some horses with Theiler’s disease (*5*,*8*). Microscopic findings in clinically affected horses with serum hepatitis consistently include widespread centrilobular to midzonal hepatocellular necrosis with hemorrhage; portal areas have mild inflammatory infiltrate, primarily monocytes and lymphocytes, and moderate bile duct proliferation (*39*,*40*). Liver enzymes, including SDH and AST, are increased several fold, and GGT is increased but often not to the same magnitude as the hepatocellular enzymes (*7*,*8*). We therefore believe that the incubation period after tetanus antitoxin administration, combined with serum chemistry and histopathologic finding in the 2 experimentally infected horses, is compatible with prior reports on equine serum hepatitis (*3*–*8*,*11*).

The virus and serologic survey findings of EqPV-H viremia in 13% of horses without biochemical evidence of liver disease suggests that most horses that become infected with EqPV-H do not develop clinical disease. This finding would be compatible with epidemiologic data on Theiler’s disease outbreaks in which clinical hepatitis develops in only 1.4%–2.2% of horses receiving equine blood products (*1*,*2*). One limitation of our study was that we could not determine the exact chronicity of infection in the horses in the serologic study and if these horses had biochemical evidence of liver disease at some prior point during infection. Although our limited study indicates low genetic diversity among EqPV-H isolated from different horses, follow-up studies that include horses living in different geographic areas are necessary to define the true prevalence and genetic diversity of EqPV-H. Why some horses develop severe and often fatal disease after EqPV-H infection and others do not also remains unknown and requires further investigation. 

Several findings in this study suggest that EqPV-H infections in horses can often be persistent. In the serologic/virus prevalence study, only 2 horses with EqPV-H antibody were virus negative, and the 13 viremic horses all had antibody. In addition, both experimentally infected horses were virus positive after 123 days, despite having high antibody levels. We have no data on the ability of antibody specific for EqPV-H to neutralize the virus or on the role the antibody might play in maintaining infection. Finally, retrospective testing of administered antitoxin and serum samples of recipient horses indicated presence of EqPV-H in all samples that tested positive for TDAV infection in our previous study (*9*). We believe that because the previous study used RNA-only viral metagenomics, the EqPV-H (a DNA virus) remained elusive.

 In summary, information from this study suggests that EqPV-H can cause serum hepatitis (Theiler’s disease) in horses. EqPV-H in horse serum or plasma products should be of concern.
